# SLC39A10 promotes malignant phenotypes of gastric cancer cells by activating the CK2-mediated MAPK/ERK and PI3K/AKT pathways

**DOI:** 10.1038/s12276-023-01062-5

**Published:** 2023-08-01

**Authors:** Xiaojuan Ren, Chao Feng, Yubo Wang, Pu Chen, Simeng Wang, Jianling Wang, Hongxin Cao, Yujun Li, Meiju Ji, Peng Hou

**Affiliations:** 1 Key Laboratory for Tumor Precision Medicine of Shaanxi Province, The First Affiliated Hospital of Xi’an Jiaotong University, 710061 Xi’an, P. R. China; 2 Department of Endocrinology, The First Affiliated Hospital of Xi’an Jiaotong University, 710061 Xi’an, P. R. China; 3 Department of Endocrinology, The Second Affiliated Hospital of Xi’an Jiaotong University, 710004 Xi’an, P. R. China; 4 Center for Translational Medicine, The First Affiliated Hospital of Xi’an Jiaotong University, 710061 Xi’an, P. R. China

**Keywords:** Gastric cancer, Prognostic markers, Mechanisms of disease, Gastric cancer

## Abstract

Solute carrier family 39 member 10 (SLC39A10) belongs to a subfamily of zinc transporters and plays a key role in B-cell development. Previous studies have reported that its upregulation promotes breast cancer metastasis by enhancing the influx of zinc ions (Zn^2+^); however, its role in gastric cancer remains totally unclear. Here, we found that SLC39A10 expression was frequently increased in gastric adenocarcinomas and that SLC39A10 upregulation was strongly associated with poor patient outcomes; in addition, we identified SLC39A10 as a direct target of c-Myc. Functional studies showed that ectopic expression of SLC39A10 in gastric cancer cells dramatically enhanced the proliferation, colony formation, invasiveness abilities of these gastric cancer cells and tumorigenic potential in nude mice. Conversely, SLC39A10 knockdown inhibited gastric cancer cell proliferation and colony formation. Mechanistically, SLC39A10 exerted its carcinogenic effects by increasing Zn^2+^ availability and subsequently enhancing the enzyme activity of CK2 (casein kinase 2). As a result, the MAPK/ERK and PI3K/AKT pathways, two major downstream effectors of CK2, were activated, while c-Myc, a downstream target of these two pathways, formed a vicious feedback loop with SLC39A10 to drive the malignant progression of gastric cancer. Taken together, our data demonstrate that SLC39A10 is a functional oncogene in gastric cancer and suggest that targeting CK2 is an alternative therapeutic strategy for gastric cancer patients with high SLC39A10 expression.

## Gastric cancer: Zinc transporter protein fuels disease progression

A zinc transporter protein that is upregulated in gastric and other cancers offers a potential treatment target. Zinc is a vital trace element in the body, and a structural and catalytic component of many enzymes. Scientists know that the upregulation of the zinc transporter protein SLC39A10 enhances the influx of zinc ions into breast and liver cancer cells, boosting proliferation and metastasis. However, the role of SLC39A10 in other cancers is unclear. Xiaojuan Ren and co-workers at Xi’an Jiaotong University in Xi’an, China, found that SLC39A10 is upregulated in human gastric cancer cells and this increase is linked to poor prognosis. SLC39A10 increased the invasiveness of gastric cancer cells and their tumorigenic potential in mouse models. The team outlined the mechanisms behind SLC39A10 activity, highlighting two key pathways and identifying a possible treatment route.

## Introduction

Gastric cancer is a common solid malignancy with a relatively poor prognosis and has become the fourth leading cause of cancer-related death worldwide^1^. Gastric cancer is a disease with high molecular and phenotypic heterogeneity. Although the diagnostic methods and treatments for gastric cancer are constantly improving, due to the insidiousness of the early symptoms and the lack of effective screening for the early diagnosis of gastric cancer, most patients with gastric cancer are initially diagnosed with advanced disease, leading to poor survival outcomes^2^. The occurrence and development of gastric cancer usually involve the dysregulation of multiple signaling pathways and cytokines resulting from abnormalities such as *TP53* mutations^3^, *RAS* and *BRAF* mutations^4^, *PIK3CA* mutations^5^, methylation-mediated inactivation of *hMLH1*^6^, and overexpression of HER2^7^, VEGF/VEGFR^8^ or inflammation-related factors such as COX-2^9^. In addition, epigenetic alterations contribute to gastric cancer occurrence and development^10^. However, the mechanism underlying gastric cancer development and progression has not been completely delineated. Thus, it is a pressing need to better understand the pathogenesis of gastric cancer, thereby developing more precise methods for early diagnosis and prognostic evaluation and more effective therapeutic strategies for this disease.

Zinc is a trace element necessary for biological activities in humans, with approximately 10% of the proteins encoded by the human genome predicted to bind to zinc^11^. As an important component, zinc constitutes the catalytic active center of various enzymes and is present in much larger quantities than other trace elements, such as copper and iron^12^. Zinc not only participates in the structure and function of various enzymes, transcription factors, cytokines and other proteins but also acts as a cellular signaling molecule to mediate physiological processes such as cell proliferation, differentiation and migration^13,14^. Thus, Zn^2+^ dyshomeostasis in the body can lead to the occurrence of various diseases^15^. There is strong evidence showing the connection between Zn^2+^ and kinase signaling. For example, Zn^2+^ is associated with ERK-dependent cell death in neurons and neuronal cell lines^16,17^. Zn^2+^ supplementation can promote the proliferation and prevent the differentiation of myogenic cells via ERK and AKT signaling^18^.

There are two important transporter families involved in the metabolic control of Zn^2+^ homeostasis: the SLC39A (solute linked carrier 39A)/ZIP (Zrt- and Irt-like proteins) family and the SLC30A (solute linked carrier 30A)/ZnT (Zn transporter) family, which participate in the transfer of Zn^2+^ into and out of cells^19^. They can activate bodily development, bone growth, immune function, endocrine regulation, protein and nucleic acid metabolism, and other physiological activities by regulating Zn^2+^ metabolism^15,20^. In addition, these two transport families not only participate in the steady-state metabolism of Zn^2+^ but also affect the occurrence and development of human diseases such as cancer and diabetes via complex mechanisms^21^. For example, as ZIP4 mediates the initial absorption of dietary zinc, patients with *ZIP4* mutations develop hereditary enteropathic acrodermatitis^22,23^. Mutational inactivation of *ZIP13*, which controls the formation of bone, tooth and connective tissue by regulating the BMP/TGF-β signaling pathway, leads to the development of Ehler-Danlos syndrome with vertebral dysplasia^24,25^. ZIP14 has been demonstrated to regulate G protein-coupled receptor signaling^26^, while ZIP8 is involved in the development of osteoarthritis and negatively regulates the activation of NF-κB^27,28^. Furthermore, SLC39A10/ZIP10 has been demonstrated to be involved in physiological and pathological processes such as immunity and tumor formation^29–31^. For example, SLC39A10 expression was found to be elevated in breast cancers and related to poor prognosis^32^, and ectopic expression of SLC39A10 enhanced breast cancer cell invasiveness and metastasis^33,34^. SLC39A10 expression on the cell membrane was proven to be sensitively regulated by the state of Zn^2+^, suggesting that SLC39A10 may be a key transporter in the maintenance of cell Zn^2+^ homeostasis in breast cancer^35^. Upregulation of SLC39A10 promoted tumor aggressiveness and was strongly associated with immune infiltration and poor patient outcomes in hepatocellular carcinoma^36,37^. SLC39A10 inhibited caspase activity by increasing Zn^2+^ influx to promote early B-cell survival in the early stages of B-cell development^38^. In addition, SLC39A10 promoted proliferation and chemoresistance via the ITGA10-mediated PI3K/AKT pathway in osteosarcoma^39^. However, the specific role of SLC39A10 in human cancers, including gastric cancer, has not been completely delineated.

Here, we found that SLC39A10 is highly expressed in gastric adenocarcinomas and observed the association of its upregulation with poor patient outcomes. Further studies revealed that SLC39A10 exerts oncogenic roles in gastric cancer cells by elevating Zn^2+^ influx, thereby activating CK2 (casein kinase 2)-related signaling pathways. In addition, we demonstrated that SLC39A10 forms a vicious feedback loop with c-Myc to promote the malignant progression of gastric cancer.

## Materials and methods

### Clinical samples

Twenty pairs of primary gastric adenocarcinomas and matched paracancerous tissues (control subjects) were collected from the First Affiliated Hospital of Xi'an Jiaotong University. Fresh tissues were fixed in 10% neutral formaldehyde, embedded in paraffin and sectioned (4 μm) for immunohistochemical (IHC) assays. All patients who did not receive any therapeutic interventions signed an informed consent form before surgery. All tissues were histologically examined by an experienced pathologist. This program was authorized by the human ethics committee and the institutional review board.

### RNA isolation and qRT‒PCR

These procedures were performed in a manner similar to that described in previous reports^40^. The relative mRNA expression of the indicated genes was normalized to *β-actin* cDNA expression. Each sample was analyzed in triplicate. Supplementary Table 1 shows the primer sequences.

### Cell culture and drug treatments

The human gastric cancer cell lines MKN45 and AGS were routinely cultured at 37 °C in RPMI 1640 medium containing 10% FBS. Cells were treated with 3.5 μM TPEN (Sigma‒Aldrich) for 24 h to chelate intracellular Zn^2+^. In certain experiments, we treated cells with 25 μM MG132 (Selleck Chemicals) for 4 h to block the ubiquitin‒proteasome pathway and 10 μM 10074-G5 (Selleck Chemicals) for 24 h to inhibit c-Myc. Additionally, we treated cells with the selective CK2 (casein kinase 2) inhibitor silmitasertib (CX-4945) (Selleck Chemicals) at a concentration of 5 μM for 24 h, as previously mentioned^41^.

### Ectopic expression and knockdown of SLC39A10

siRNA oligonucleotides specifically targeting SLC39A10 and the control siRNA were purchased from RiboBio. According to the manufacturer’s instructions, MKN45 and AGS cells at 50% confluence were transfected with X-tremeGENE (Invitrogen). Two of the three siRNAs with the highest knockdown efficiency were selected for the following experiment.

The lentivirus expressing SLC39A10, the lentivirus expressing shRNA targeting SLC39A10 and the corresponding control lentiviruses were purchased from HanBio Biotechnology Co., Ltd. The lentivirus expressing c-Myc and its control lentivirus were purchased from GeneChem Co., Ltd. (Shanghai. China). The sequences of the corresponding constructs are presented in Supplementary Table 2.

### In vitro functional studies

A series of in vitro functional experiments, including cell proliferation, colony formation, cell cycle, apoptosis and cell migration/invasion assays, were performed in a manner similar to that described in previous reports^42^.

### Western blotting analysis

The procedure was performed in a manner similar to that described in previous reports^42^. The antibodies used in this study are listed in Supplementary Table 3.

### Dual-luciferase reporter assay

To construct the luciferase reporter plasmid (pGL3-SLC39A10-Luc), the promoter region of the SLC39A10 gene was inserted into the preprocessed luciferase vector pGL3-Basic (Promega Corp., WI) after amplification using the primers shown in Supplementary Table 4. Sanger sequencing was then carried out to verify the construct. The pGL3-Basic-Luc or pGL3-SLC39A10-Luc plasmid and the pRL-TK (internal control, Promega) plasmid were cotransfected into c-Myc-overexpressing cells and control cells to determine the regulatory effect of c-Myc on the *SLC39A10* promoter. After a 48-h transfection, we harvested cells and used a dual-luciferase reporter assay system (Promega) to measure the luciferase activity according to the manufacturer’s instructions. The ratio of firefly luciferase intensity to Renilla luciferase intensity indicates the relative luciferase activity. Each experiment was performed three times.

### Chromatin immunoprecipitation (ChIP) assay

A ChIP assay was conducted to assess the binding of c-Myc to its target DNA in a manner similar to that described in previous reports^19^. A rabbit polyclonal antibody against c-Myc (Santa Cruz, sc-764) was used for this assay. The primer sequences used for the ChIP‒qPCR assay are presented in Supplementary Table 5, and three replicates were analyzed in each assay.

### Measurement of intracellular Zn^2+^

The Zn^2+^ probe FluoZin™-3 (Molecular Probes, Invitrogen) was used to measure the intracellular free Zn^2+^ concentration by flow cytometry or confocal microscopy (Leica). For flow cytometric analysis, the indicated cells were trypsinized, centrifuged and washed. Next, the cells were incubated with 1 μM FluoZin™-3 for 50 min at 37 °C, and the fluorescence intensity was then measured. For confocal microscopy analysis, cells were seeded in confocal Petri dishes (MatTek Corporation) and then transfected with the overexpression vector or empty vector. The cell culture medium was removed after 48 h, and the cells were then incubated with 1 μM FluoZin™-3 at 37 °C for 50 min. Next, the cells were rinsed, and the fluorescence intensity was measured at 516 nm emission and 494 nm excitation after a 30-min incubation.

### Cycloheximide (CHX) chase assay

SLC39A10-knockdown gastric cancer cells and the corresponding control cells were treated with 10 µM CHX (MP Biomedicals), a new protein synthesis inhibitor, for the indicated time to inhibit de novo protein synthesis. After harvesting, the cells were lysed for western blotting analysis.

### Protein expression, refolding and purification

The human CK2β sequence (Gene ID: 1460, CSNK2B) was cloned and then inserted into the pET-28a (+) plasmid. *Escherichia coli* BL21 (DE3) cells were then transformed with the resulting pET-28a (+)-HIS-CK2β plasmid. Next, BL21 cells were cultured overnight and diluted in fresh LB (1:100); 0.5 mM IPTG was then added when the OD_600nm_ value was approximately 0.6, and culture was continued at 28 °C for 12 h. Sonication with PBS buffer was performed to lyse the bacterial cells after collection by centrifugation. Inclusion bodies were washed three times with 1 M urea and then solubilized in 8 M urea at 4 °C overnight. Solubilized recombinant CK2β was further purified using His-tag purification resins (Beyotime Biotechnology) and then refolded by dilution in buffer containing 5 mM DTT and 20 mM Tris (pH 7.2) with or without 4 μM ZnSO_4_. Next, the solution was subjected to four rounds of dialysis at 4 °C with this buffer prior to filtration through a 0.2-μm filter. The solubilized supernatants were then loaded onto SDS‒PAGE gels, and the gels were subjected to Coomassie blue staining.

### Circular dichroism (CD) spectroscopy assay

CD and thermal denaturation assays were performed in a manner similar to that described in previous reports^43^.

### Animal studies

Fifteen female athymic nude mice (4 weeks old) were obtained from SLAC Laboratory Animal Co., Ltd. We then randomly divided these mice into three groups and established tumor xenografts by subcutaneously implanting 1 × 10^6^ SLC39A10-overexpressing MKN45 cells or control cells into the right axillae of the nude mice. Three days after injection, CX-4945 or vehicle was administered at 50 mg/kg twice daily by oral gavage. During the treatment period, we measured the tumor size every 2 days and calculated tumor volume using a formula (length × width^2^/2). After 15 days of consecutive daily dosing, these mice were sacrificed, and the xenograft tumors were then isolated and weighed. In addition, tumor tissues were paraffin-embedded and sectioned. All animal experimental procedures were approved by the Laboratory Animal Center of Xi'an Jiaotong University.

### Immunohistochemical (IHC) analysis

IHC analysis was performed to assess the levels of phosphorylated AKT (p-AKT; Ser473, Ser129 and Thr308), phosphorylated ERK1/2 (p-ERK1/2), c-Myc and Ki-67 in the xenograft tumors as previously described^44^.

### Statistical analysis

Gene expression in cancer tissues and control subjects was compared by an unpaired *t* test, while gene expression in paired samples was compared by a paired *t* test. The Kaplan‒Meier method was used to construct survival curves, and differences were tested by the log-rank test. In addition, one-way ANOVA with Dunnett’s post hoc test and two-way ANOVA with the Bonferroni correction were used to compare the data. All statistical analyses were calculated with SPSS statistical software (16.0, Chicago, IL). *P* < 0.05 was considered statistically significant.

## Results

### SLC39A10 expression is elevated in gastric adenocarcinomas and strongly associated with poor patient outcomes

**Fig. 1:**
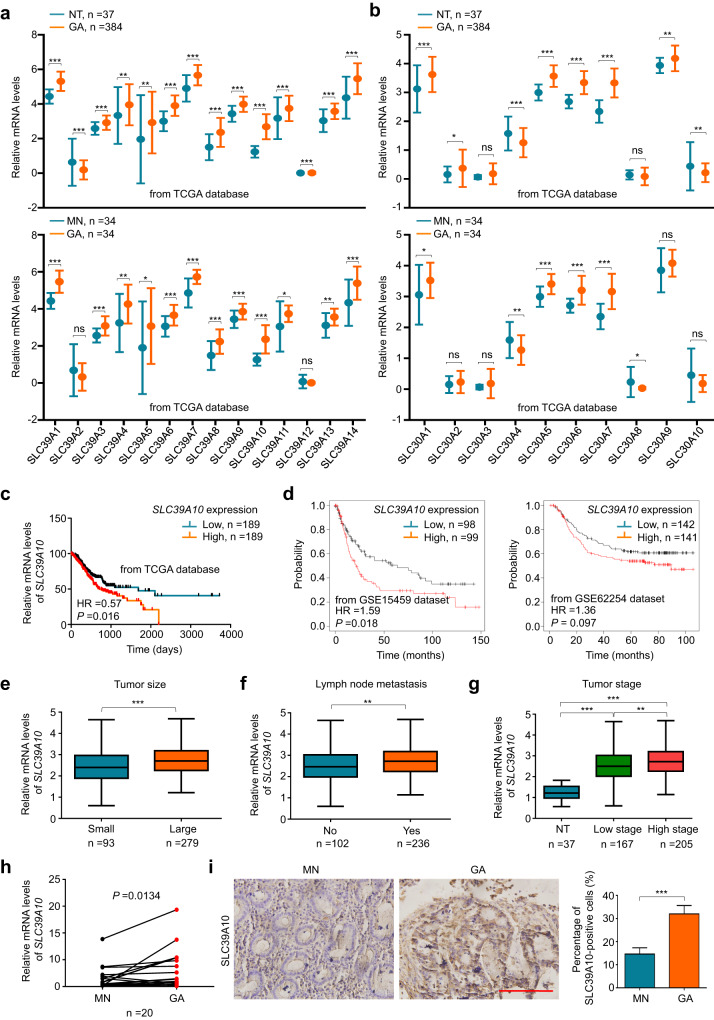
Correlation of elevated expression of SLC39A10 with poor clinical outcomes in gastric cancer patients. **a** The mRNA expression of SLC39A family members in gastric adenocarcinomas (GAs) and normal gastric tissues (NTs) or the matched noncancerous tissues (MNs) (data from the TCGA RNA-Seq dataset). **b** The mRNA expression of SLC30A family members in GAs and NTs or MNs (data from the TCGA RNA-Seq dataset). A significant association of increased expression of SLC39A10 with poor patient survival in the TCGA dataset (**c**) and two GEO datasets (GSE15459 and GSE62254) (**d**) was found using Kaplan‒Meier Plotter. The box plots show the expression level of *SLC39A10* in GA tumors of different sizes (**e**), lymph node metastasis statuses (**f**) and stages (**g**) (data from the TCGA database). The horizontal lines indicate the median value. **h** qRT‒PCR results showing the mRNA expression of *SLC39A10* in GAs and the matched noncancerous gastric tissues (MN; n = 20). The *SLC39A10* expression level was normalized to the *18* *S* rRNA level. The data were compared using a two-tailed paired *t* test. **i** Representative images showing immunohistochemical (IHC) staining of SLC39A10 on histologic slides of GAs and MNs in the left panel. The percentage of SLC39A10-positive cells is shown in the right panel. The error bars indicate the SDs. *P < 0.05; **P < 0.01; ***P < 0.001. Scale bar, 200 µm.

We first analyzed the mRNA expression of SLC39A and SLC30A family members in gastric adenocarcinoma tissues and normal gastric tissues or the matched normal gastric tissues using the Cancer Genome Atlas (TCGA) RNA-Seq dataset. The results showed that the expression of *SLC39A1*, *SLC39A3*, *SLC39A4*, *SLC39A5*, *SLC39A6*, *SLC39A7*, *SLC39A8*, *SLC39A9*, *SLC39A10*, *SLC39A11*, *SLC39A13* and *SLC39A14* was significantly upregulated in gastric adenocarcinoma tissues compared with both normal gastric tissues and matched normal gastric tissues (Fig. 1a). In addition, we found that the expression levels of *SLC30A1*, *SLC30A5*, *SLC30A6* and *SLC30A7* were significantly elevated, while the expression level of *SLC30A4* was significantly decreased in gastric adenocarcinoma tissues compared with both normal gastric tissues and matched normal gastric tissues (Fig. 1b). Next, we investigated the correlation of these differentially expressed genes with the prognosis of gastric adenocarcinoma patients using the TCGA database and found a significant relationship between elevated expression of only *SLC39A10* and poor patient survival (Fig. 1c). This relationship was also supported by analysis of the GSE15459 and GSE62254 datasets (Fig. 1d). We also observed a positive correlation of *SLC39A10* expression with tumor size (Fig. 1e), lymph node metastasis (Fig. 1f), and tumor stage (Fig. 1g) using the TCGA database. Next, we further analyzed SLC39A10 expression in different histologic types of gastric cancer using the TCGA database and found that its expression was higher in cancerous tissues than in noncancerous tissues in most diffuse- and intestinal-type gastric adenocarcinomas, except for some types that were not analyzed due to a lack of control subjects (Supplementary Fig. 1). In addition, we selected 20 pairs of gastric adenocarcinoma tissues, which were diagnosed by H&E staining (Supplementary Fig. 2), and the matched noncancerous tissues and examined SLC39A10 expression in these tissues by qRT‒PCR analysis and IHC staining. The results further confirmed the increased expression of SLC39A10 in cancer tissues compared with control tissues (Fig. 1h, i).

### SLC39A10 is identified as a direct downstream target of c-Myc

To determine the mechanism of SLC39A10 upregulation, we used an online tool (http://www.genecards.org and https://jaspar.genereg.net/) to predict potential transcription factors with binding sites within the *SLC39A10* promoter and found that the *SLC39A10* promoter region contains multiple binding sites for c-Myc, which has been extensively studied in human cancers^45^ and gastric tumorigenesis^46^. Thus, we next determined the relationship between c-Myc expression and *SLC39A10* expression in gastric cancers using an online tool (http://gepia2.cancer-pku.cn/) and demonstrated a positive association between these mRNA expression levels (Supplementary Fig. 3), suggesting that c-Myc may regulate *SLC39A10* transcription.

**Fig. 2:**
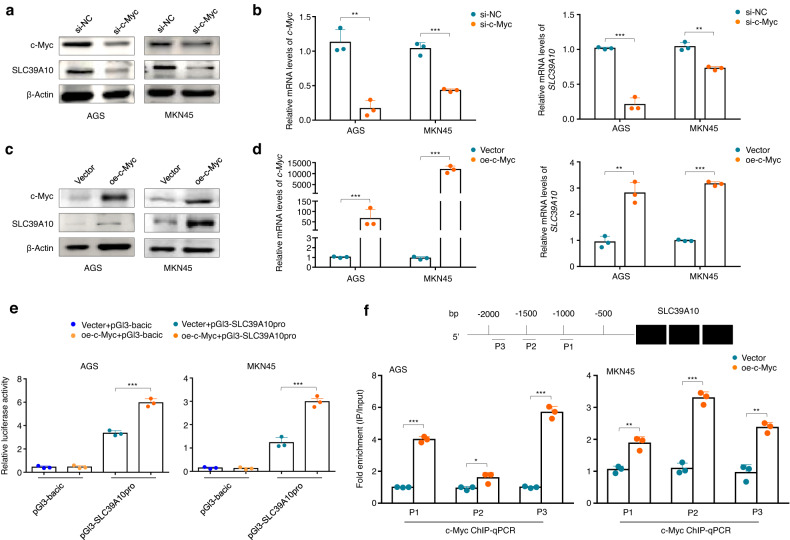
Identification of *SLC39A10* as a downstream target of c-Myc in gastric cancer cells. Western blotting (**a**) and qRT‒PCR (**b**) analysis results showing the effect of c-Myc knockdown on SLC39A10 expression in the gastric cancer cell lines AGS and MKN45. Western blotting (**c**) and qRT‒PCR (**d**) analysis results showing the effect of ectopic expression of c-Myc on SLC39A10 expression in AGS and MKN45 cells. Representative western blottings from three independent western blotting analyses are shown. β-Actin was used as the normalization control for western blotting and qRT‒PCR. **e** The dual-luciferase reporter assay results showing the effect of ectopic expression of c-Myc on the promoter activity of *SLC39A10* in AGS and MKN45 cells, with empty vector or control lentivirus as the controls. All the ratios of firefly/Renilla luciferase activity are expressed as the means ± SDs. **f** AGS and MKN45 cells expressing c-Myc and the corresponding control cells were subjected to ChIP‒qPCR assays using the corresponding primary antibodies. P1–P3 indicate three different regions of the *SLC39A10* promoter (P1: −1044/−941; P2: −1518/−1273; P3: −-1939/−1671) (upper panel). Fold enrichment values are expressed as the means ± SDs (lower panels). *P < 0.05; **P < 0.01; ***P < 0.001.

To further determine the regulatory role of c-Myc in *SLC39A10* expression, we knocked down c-Myc in AGS and MKN45 cells and found that SLC39A10 knockdown obviously decreased the protein and mRNA expression of SLC39A10 compared with the control cells (Fig. 2a, b). Conversely, overexpressing c-Myc in these two cell lines significantly elevated SLC39A10 expression compared with the control cells (Fig. 2c, d). Next, we constructed the luciferase reporter plasmid pGL3-SLC39A10-Luc (−2000/ + 1) by inserting the promoter region of *SLC39A10* into the pGL3-Basic luciferase plasmid and cotransfected c-Myc-overexpressing AGS and MKN45 cells and control cells with the pGL3-SLC39A10-Luc or pGL3-Basic-Luc and pRL-TK plasmids. The results showed that c-Myc overexpression markedly increased the promoter activity of *SLC39A10* compared with the control cells (Fig. 2e). In addition, to determine whether c-Myc regulated the promoter activity of *SLC39A10* by binding directly to its promoter region, we employed a ChIP‒qPCR assay in c-Myc-overexpressing AGS and MKN45 cells and control cells using a specific anti-c-Myc antibody and primers specific to three different regions within the *SLC39A10* promoter (Fig. 2f, upper panel). As expected, all three fragments within the *SLC39A10* promoter (P1: −1044/-941; P2: −1518/−1273; P3: −1939/−1671) were distinctly enriched in c-Myc-overexpressing cells compared with control cells. Taken together, these data support the idea that *SLC39A10* is a direct downstream target of c-Myc.

### SLC39A10 accelerates gastric cancer cell growth and invasiveness

**Fig. 3:**
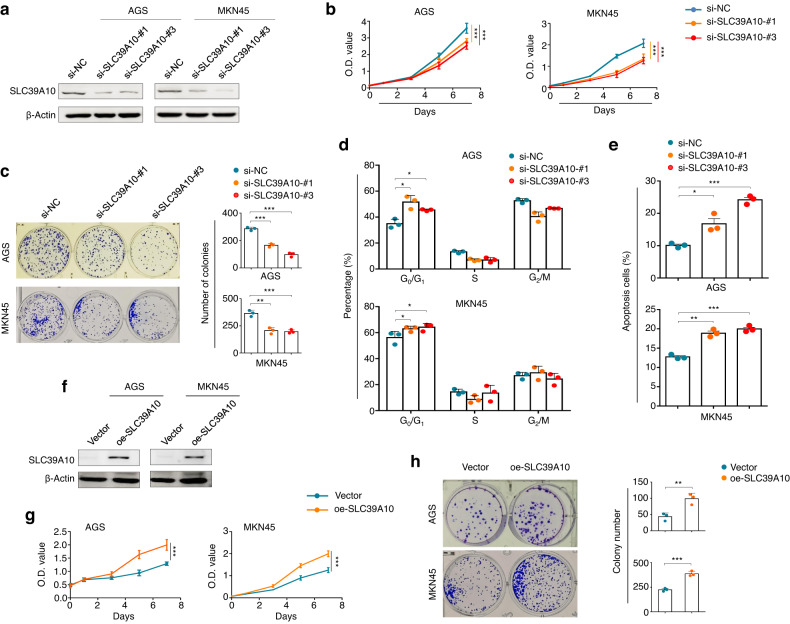
The effect of SLC39A10 on proliferation, colony formation, the cell cycle distribution and apoptosis in gastric cancer cells. **a** SLC39A10 knockdown in AGS and MKN45 cells by using two different siRNAs (si- SLC39A10-#1 and -#3) was confirmed by western blotting analysis. β-Actin was used as a loading control. **b** MTT assay showing the inhibitory effect of SLC39A10 knockdown on cell proliferation in AGS and MKN45 cells. **c** The inhibitory effect of SLC39A10 knockdown on colony formation in the indicated cells. Representative images showing colony formation are provided in the left panels, and the quantitative analysis results are shown in the right panel. AGS and MKN45 cells were transiently transfected with the indicated siRNAs. The cell cycle distribution (**d**) and apoptosis (**e**) were then analyzed by flow cytometry. **f** Ectopic expression of SLC39A10 in AGS and MKN45 cells was confirmed by western blotting analysis. β-Actin was used as a loading control. The promoting effect of ectopic expression of SLC39A10 on cell proliferation (**g**) and colony formation (**h**). The data are presented as the means ± SDs. *P < 0.05; **P < 0.01; ***P < 0.001.

To determine the functions of SLC39A10 in gastric cancer cells, we first knocked down SLC39A10 in AGS and MKN45 cells using two different siRNAs (si- SLC39A10-#1 and -#3) specifically targeting SLC39A10 and verified their inhibition efficiency by qRT‒PCR (Supplementary Fig. 4a) and western blotting (Fig. 3a). The results of functional studies showed that SLC39A10 knockdown strongly suppressed gastric cancer cell proliferation (Fig. 3b) and colony formation (Fig. 3c) in comparison with the control cells. We further investigated the impacts of SLC39A10 knockdown on the cell cycle distribution and apoptosis in gastric cancer cells. The results showed that the cell cycle was obviously arrested at the G_0_/G_1_ phase boundary in SLC39A10-knockdown AGS and MKN45 cells compared with control cells (Fig. 3d). In addition, SLC39A10 knockdown dramatically induced an increase in the percentage of apoptotic cells in comparison with the control group (Fig. 3e). Conversely, overexpressing SLC39A10 in AGS and MKN45 cells enhanced cell proliferation and colony formation compared with the control cells, as expected (Supplementary Fig. 4b and Fig. 3f–h).

Next, we assessed the impact of SLC39A10 on the migration and invasion of AGS and MKN45 cells. The results showed that SLC39A10-knockdown cells showed relatively weak cell migration (Supplementary Fig. 5a) and cell invasion (Supplementary Fig. 5b) abilities in comparison with control cells. In contrast, overexpressing SLC39A10 clearly enhanced the cell migration/invasion potential relative to the control cells (Supplementary Fig. 5c, d). These results further indicate the tumor-promoting role of SLC39A10 in gastric cancer cells.

### SLC39A10 increases the phosphorylation of ERK and AKT by enhancing Zn^2+^ influx

**Fig. 4:**
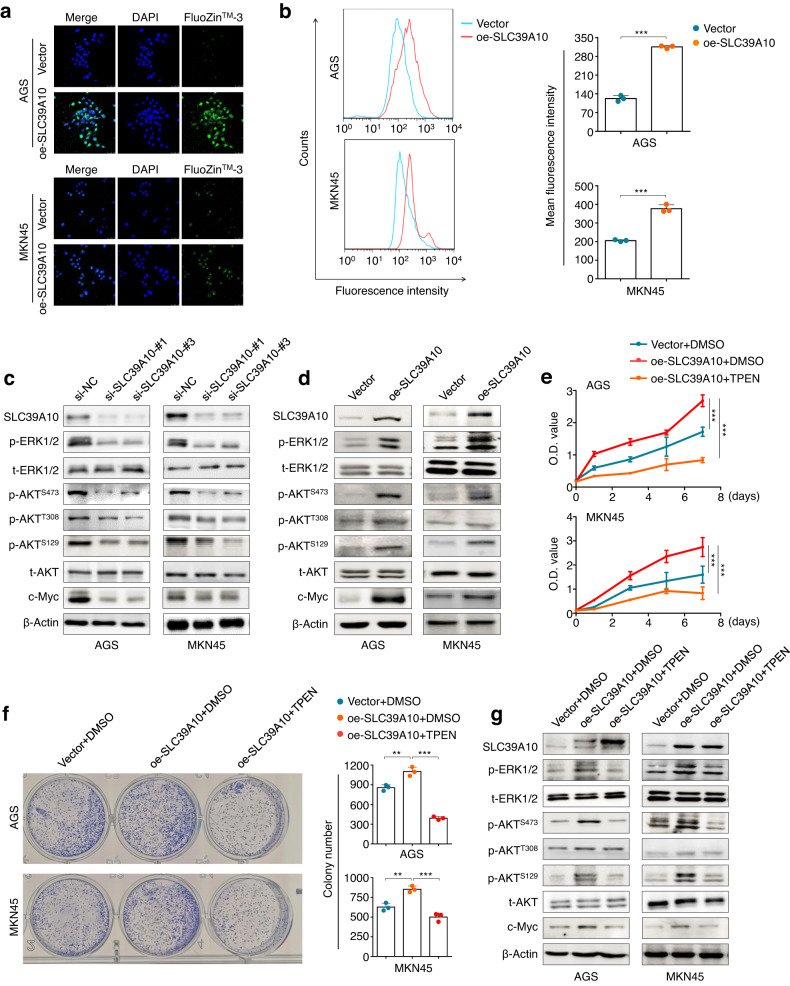
Activating effect of SLC39A10 on the MAPK/ERK and PI3K/AKT pathways via an increase in the intracellular Zn^2+^ concentration. **a** Representative images showing free intracellular Zn^2+^ in SLC39A10-overexpressing AGS and MKN45 cells and control cells under a confocal microscope (left panel). Blue indicates staining of nuclei. Green indicates staining of free intracellular Zn^2+^. Scale bars, 200 μm. **b** The free intracellular Zn^2+^ content, as measured by flow cytometry, is shown in the left panel, and the right panel shows the statistical results. Western blotting analysis showing the effect of SLC39A10 knockdown (**c**) and overexpression (**d**) on MAPK/ERK and PI3K/AKT pathway activity and c-Myc expression in AGS and MKN45 cells. β-Actin was used as a loading control. SLC39A10-overexpressing AGS and MKN45 cells and control cells were treated with 3.5 μM TPEN or vehicle for 24 h, and the effects of these treatments on cell proliferation (**e**), colony formation (**f**) and MAPK/ERK and PI3K/AKT pathway activity (**g**) were evaluated by MTT, colony formation and western blotting assays. β-Actin was used as a loading control. The data are presented as the means ± SDs. *P < 0.05; **P < 0.01; ***P < 0.001.

SLC39A10 is similar in structure and function to other Zn^2+^ transporter subfamily members, which transport Zn^2+^ across membranes and maintain intracellular Zn^2+^ concentrations^47^. To prove this assertion, we first determined the effect of SLC39A10 on intracellular Zn^2+^ levels and demonstrated using laser scanning confocal microscopy that ectopic expression of SLC39A10 significantly elevated the Zn^2+^ fluorescence intensity in the cytoplasm of AGS and MKN45 cells in comparison with the control cells (Fig. 4a). This was also supported by the results of flow cytometric analysis, which showed a significant increase in the mean fluorescence intensity in SLC39A10-overexpressing cells compared with control cells (Fig. 4b). These results further confirm that SLC39A10 can promote Zn^2+^ influx.

A previous study demonstrated that Zn^2+^ influx can activate the MAPK/ERK and PI3K/AKT cascades^48^, which play a vital role in gastric tumorigenesis^49,50^. Thus, we decided to determine the impact of SLC39A10 on the activity of these two pathways. The results showed that SLC39A10 knockdown in AGS and MKN45 cells strongly suppressed ERK1/2 phosphorylation and AKT phosphorylation at Ser473 and Ser129 but slightly decreased AKT phosphorylation at Thr308; moreover, the expression of c-Myc, a major target of these two pathways, was correspondingly inhibited by SLC39A10 knockdown and promoted by SLC39A10 overexpression (Fig. 4c, d). To further confirm whether SLC39A10 activates the MAPK/ERK and PI3K/AKT signaling pathways by altering intracellular Zn^2+^ homeostasis, we treated SLC39A10-overexpressing AGS and MKN45 cells and control cells with 3.5 μM TPEN, a highly selective Zn^2+^ chelator, for 24 h. As expected, we found that the promoting effects of SLC39A10 on cell proliferation (Fig. 4e) and colony formation (Fig. 4f) were effectively reversed upon TPEN treatment. Accordingly, TPEN treatment also reversed the enhancing effect of SLC39A10 overexpression on the phosphorylation of ERK1/2 and AKT and expression of c-Myc (Fig. 4g). Taken together, these data indicate that SLC39A10 activates the MAPK/ERK and PI3K/AKT signaling pathways by enhancing Zn^2+^ influx.

### Zn^2+^ interacts with the binding domains of two CK2β monomers to form a stable dimer structure

**Fig. 5:**
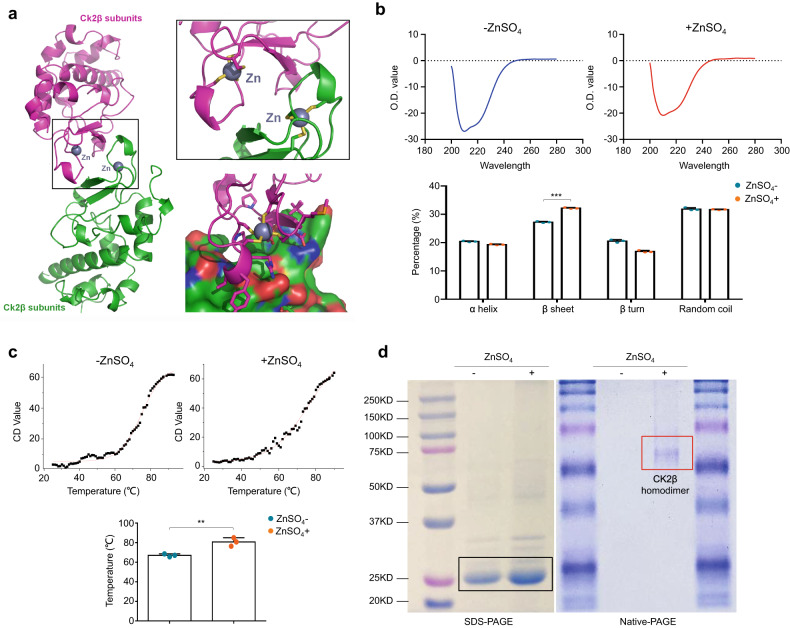
The effect of Zn^2+^ on the secondary structure of the CK2β protein. **a** A structural model of the CK2β homodimer is shown in the left panel, and an enlarged view of the CK2β homodimer binding interface and a detailed map of interactions at the CK2β homodimer binding interface are shown in the right panel. **b** Protein conformation analysis by circular dichroism (CD) spectroscopy in the presence or absence of Zn^2+^ is shown in the upper panels, and the precise data are shown in the lower panel. **c** CK2β protein stability in the presence and absence of Zn^2+^ was confirmed by CD spectroscopy combined with a thermal denaturation assay (upper panels), and the quantitative analysis of the results is shown in the lower panel. **d** SDS‒PAGE, native PAGE and Coomassie blue staining showing monomeric (black box) and homodimeric (red box) CK2β proteins in the presence and absence of Zn^2+^. Data are presented as the means ± SDs. **P < 0.01; ***P < 0.001.

ERK and AKT have been demonstrated to be downstream effectors of CK2. For example, CK2 can phosphorylate ERK at Ser244 and Ser246 to promote the intracellular transport of ERK and the subsequent performance of its transcriptional function^51^. Moreover, AKT is directly phosphorylated at Ser129 by CK2, while Ser129 phosphorylation of AKT can increase the phosphorylation level of AKT at Ser473 and Thr308^52–54^. In addition, we found that the function of CK2 might be highly dependent on Zn^2+^ by analysis of data in the PDB (Protein Data Bank). In detail, CK2 exists as a tetramer or higher molecular weight oligomer composed of catalytic CK2α subunits and noncatalytic regulatory CK2β subunits. In particular, the homodimer formed by two β regulatory subunits is the key to the function of the CK2 protein. Further specific analysis of CK2β homodimers revealed that two CK2β monomers interacted with the stable binding domain of Zn^2+^ to form a stable dimeric structure (Fig. 5a).

To further show the important effect of Zn^2+^ on the conformation and function of CK2, bacterially expressed CK2β protein was purified and verified by Coomassie blue staining (Supplementary Fig. 6). Moreover, protein conformation analysis was performed by circular dichroism (CD) spectroscopy in the presence or absence of Zn^2+^. The results showed that the addition of Zn^2+^ obviously increased the percentage of β-sheets, consistent with the high content of β-sheets in the zinc finger structure^55^, indicating the possible formation of the zinc finger structure (Fig. 5b). In addition, the results of CD thermal denaturation assays revealed that the melting temperature (Tm) was increased in the presence of Zn^2+^ (Fig. 5c), suggesting that the protein structure is more stable in the presence of Zn^2+^ than in the absence of Zn^2+^. In addition, SDS‒PAGE showed that there were only CK2β monomers in either the presence or absence of Zn^2+^, while native PAGE and Coomassie blue staining indicated that the CK2β protein formed a dimeric structure in the presence of Zn^2+^ (Fig. 5d). Collectively, these data indicate that Zn^2+^ interacts with the binding domains of two CK2β monomers to form a stable dimeric structure, thereby enhancing the function of CK2.

### SLC39A10 enhances the protein stability of CK2β and holoenzyme activity of CK2

**Fig. 6:**
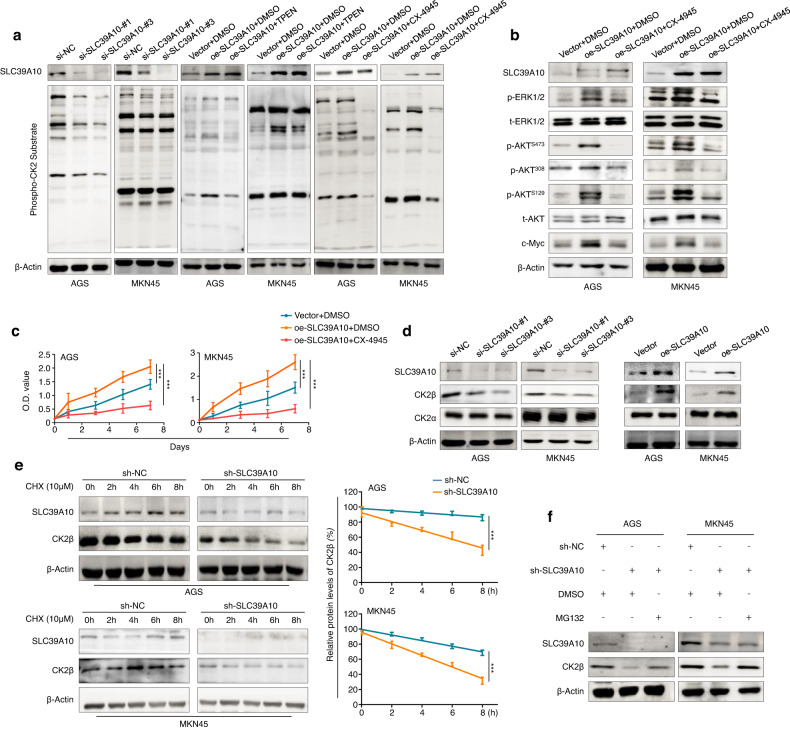
The enhancing effect of SLC39A10 on the protein stability of CK2β and the holoenzyme activity of CK2. **a** The effect of SLC39A10 knockdown on the holoenzyme activity of CK2 was evaluated by western blotting analysis (first two panels). In addition, SLC39A10-overexpressing AGS and MKN45 cells and control cells were treated with 3.5 μM TPEN, 5 μM silmitasertib (CX-4945, a selective CK2 inhibitor) or vehicle for 24 h, and the holoenzyme activity of CK2 was then quantified by western blotting analysis (the last four panels). β-Actin was used as a loading control. **b** SLC39A10-overexpressing AGS and MKN45 cells were treated with 5 μM CX-4945 for 24 h, and western blotting analysis was then performed to test the effect of CX-4945 treatment on MAPK/ERK and PI3K/AKT pathway activity. β-Actin was used as a loading control. **c** An MTT assay was carried out to evaluate cell proliferation in SLC39A10-overexpressing AGS and MKN45 cells and control cells with the indicated treatments. **d** The effect of SLC39A10 knockdown or overexpression on the protein expression of CK2α and CK2β was evaluated by western blotting analysis. β-Actin was used as a loading control. **e** SLC39A10-knockdown AGS and MKN45 cells and control cells were treated with 10 µM CHX for the indicated times, and CK2β protein expression was then evaluated by western blotting analysis (left panels). The band intensity of CK2β was normalized to that of β-Actin, and this value was then normalized to the corresponding value in the control cells (right panel). **f** The indicated cells were treated with 25 μM MG132 (a proteasome inhibitor) for 3 h, and the protein expression of SLC39A10 and CK2β was evaluated by western blotting analysis. β-Actin was used as a loading control. Data are presented as the means ± SDs. ***P < 0.001.

As mentioned above, we hypothesized that SLC39A10 may enhance the holoenzyme activity of CK2 by increasing intracellular Zn^2+^ levels, thereby activating downstream signaling pathways of CK2. To prove this, we first measured CK2 activity by analyzing the phosphorylation of CK2 substrates using an anti-phospho CK2 substrate antibody. This antibody has been proven to recognize the CK2 consensus sequence pS/pT-D-X-E, in which pS/pT indicate Ser/Thr residues phosphorylated by CK2^56^. As shown in Fig. 6a, SLC39A10 knockdown strongly decreased CK2 activity, as reflected by the obvious decrease in the phosphorylation levels of CK2 substrates in SLC39A10-knockdown AGS and MKN45 cells in comparison with the corresponding control cells. Conversely, ectopic expression of SLC39A10 increased CK2 activity, while this effect was effectively reversed by treatment with TPEN and the selective CK2 inhibitor silmitasertib (CX-4945) (Fig. 6a). Correspondingly, CX-4945 treatment also reversed the promoting effect of SLC39A10 overexpression on the phosphorylation of ERK1/2 and AKT and expression of c-Myc in AGS and MKN45 cells (Fig. 6b). Next, we treated these two cell lines with 5 μM CX-4945 for 24 h and demonstrated that CX-4945 treatment effectively reversed the enhancing effect of SLC39A10 overexpression on cell proliferation (Fig. 6c).

Further studies revealed that SLC39A10 knockdown in AGS and MKN45 cells did not greatly change the protein expression of CK2α but dramatically decreased the protein expression of CK2β compared with the control cells and that SLC39A10 overexpression had the opposite effects (Fig. 6d). However, unlike the results of western blotting analysis, the results of qRT‒PCR analysis showed that knockdown or ectopic expression of SLC39A10 did not affect the mRNA level of CK2β compared with the control cells (Supplementary Fig. 7a, b), suggesting that SLC39A10 posttranscriptionally regulates CK2β expression. To verify this, we treated SLC39A10-knockdown AGS and MKN45 cells and control cells with a new protein synthesis inhibitor, CHX. The results showed that SLC39A10 knockdown accelerated the protein degradation of CK2β compared with the control cells (Fig. 6e), while the ubiquitin proteasome inhibitor MG132 reversed the inhibitory effect of SLC39A10 knockdown on the protein expression of CK2β (Fig. 6f). These data indicate that SLC39A10 activates the MAPK/ERK and PI3K/AKT signaling pathways by enhancing the protein stability of CK2β and the holoenzyme activity of CK2.

### SLC39A10 accelerates tumor growth in nude mice

**Fig. 7:**
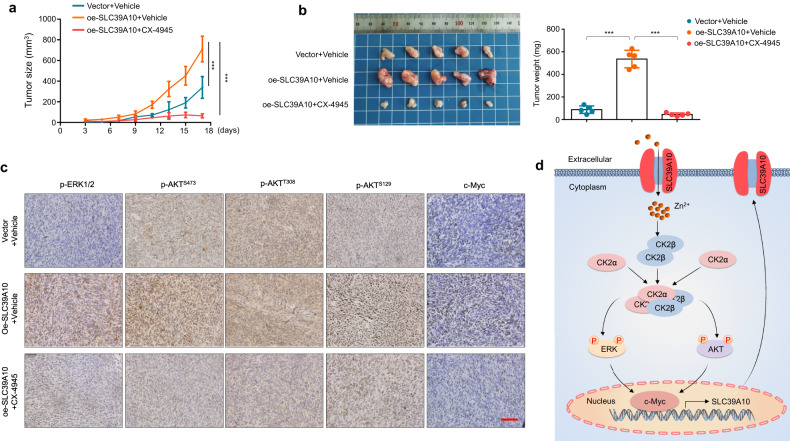
The effect of SLC39A10 knockdown or targeting CK2 on tumor growth in nude mice. **a** Growth curves of SLC39A10-overexpressing xenograft tumors and control tumors subjected to the indicated treatments (*n* = 5/group). Day 0 represents the time point of tumor cell injection, and Day 3 represents the beginning of dosing by oral gavage. **b** The left and right panels show the photographs of harvested tumors and the mean tumor weights from the indicated groups, respectively. **c** Representative sections from the indicated tumors were subjected to IHC staining using the corresponding antibodies. Scale bar, 200 μm. **d** A simple model of SLC39A10 forming a vicious loop with c-Myc to promote malignant phenotypes of gastric cancer cells.

The tumorigenic potential of SLC39A10 was evaluated in nude mice. The results showed that xenograft tumors formed by SLC39A10-overexpressing MKN45 cells grew faster and exhibited a significant increase in tumor weight in comparison with the tumors formed by control cells, while CX-4945 treatment effectively reversed the effect of SLC39A10 overexpression (Fig. 7a, b). As mentioned above, we found a positive association of SLC39A10 expression with lymph node metastasis in the TCGA database but failed to find visible metastatic loci in the major organs of mice implanted with SLC39A10-overexpressing cells (data not shown). Next, we conducted an IHC assay to determine the proliferative ability of the above xenograft tumors by evaluating the protein level of Ki-67, a proliferation marker. The results showed that SLC39A10-overexpressing tumors exhibited a significant increase in the percentage of Ki-67-positive cells compared with control tumors, and this effect was reversed by CX-4945 treatment (Supplementary Fig. 8).

We also observed increased levels of ERK1/2 phosphorylation and AKT phosphorylation at Ser473, Thr308 and Ser129 in SLC39A10-overexpressing tumors compared with control tumors, accompanied by elevated expression of c-Myc (Fig. 7c). These effects were also reversed by CX-4945 treatment (Fig. 7c). However, we did not find any effect of CX-4945 on SLC39A10 levels in xenograft tumor tissues by IHC staining (Supplementary Fig. 9), consistent with the data shown in Fig. 6b. This finding seems to contradict our conclusion. Our data suggest that CK2 can promote *SLC39A10* transcription by upregulating c-Myc. However, in fact, treatment with the CK2 inhibitor CX-4945 did not affect SLC39A10 expression. The possible explanations are as follows: (1) CK2 has too many substrates; thus, its regulation of SLC39A10 is complicated and indirect, unlike that of c-Myc, which directly regulates *SLC39A10* transcription. (2) CK2 inhibitors may have off-target effects.

To determine the therapeutic potential of SLC39A10 inhibition in gastric cancer, we stably knocked down SLC39A10 in MKN45 cells via a lentivirus system and used SLC39A10-knockdown MKN45 cells and control cells to establish a xenograft tumor model. The results showed that SLC39A10 knockdown significantly suppressed tumor growth and reduced tumor weight and the number of Ki-67‑positive cells compared with those in control animals (Supplementary Fig. 10). These data not only validate the tumorigenic potential of SLC39A10 in gastric cancer but also suggest that SLC39A10 inhibition may be a potential therapeutic strategy for gastric cancer. As mentioned above, *SLC39A10*, a downstream target of c-Myc, in turn, also upregulated c-Myc expression, thereby forming a vicious feedback loop to promote the malignant progression of gastric cancer, implying that inhibition of c-Myc may attenuate the tumor-promoting effect of SLC39A10. Thus, we treated SLC39A10-overexpressing AGS and MKN45 cells and the corresponding control cells with 10 μM 10074-G5, a c-Myc inhibitor, for 24 h. As expected, we found that the promoting effect of SLC39A10 overexpression on cell proliferation and colony formation was effectively reversed by 10074-G5 (Supplementary Fig. 11).

In summary, a simple model is proposed to clarify the mechanism underlying the tumor-promoting role of SLC39A10 in gastric cancer (Fig. 7d). Briefly, elevated expression of SLC39A10 in gastric cancer cells promotes Zn^2+^ efflux, thereby increasing intracellular Zn^2+^ levels and promoting the formation of CK2β dimers, which in turn enhances the protein stability of CK2β and the holoenzyme activity of CK2. This results in overactivation of the downstream MAPK/ERK and PI3K/AKT pathways and increased expression of c-Myc. In addition, c-Myc in turn upregulates the expression of *SLC39A10* at the transcriptional level by directly binding to its promoter, forming a vicious loop promoting the malignant progression of gastric cancer.

## Discussion

SLC39A10, an important member of the Zn^2+^ transporter family, has been reported to promote the malignant progression of breast cancer and early B-cell survival in the early stages of B-cell development^35,38^. However, its role in human cancers, including gastric cancer, has not been fully delineated. The present study provided strong evidence to support the oncogenic role of SLC39A10 in gastric cancer. We first demonstrated that SLC39A10 was frequently upregulated in gastric cancers in comparison with control subjects, and its expression was positively related to more aggressive phenotypes and poor patient survival. In addition, a series of functional studies in gastric cancer cells demonstrated that SLC39A10 promoted cell growth in vitro and in vivo, enhanced cell migration and invasion and induced cell cycle arrest and apoptosis. In addition, we clarified the mechanism of increased expression of SLC39A10 in gastric cancers by identifying SLC39A10 as a direct target of c-Myc using ChIP and dual-luciferase reporter assays.

To fully comprehend how SLC39A10 performs its oncogenic function in gastric cancer cells, we first assessed its impact on intracellular Zn^2+^ homeostasis in two gastric cancer cell lines. The results indicated that SLC39A10 overexpression led to a significant increase in the intracellular Zn^2+^ concentration in the cytoplasm of cancer cells, and there is a study demonstrating that dysregulated Zn^2+^ homeostasis can induce the activation of some major signaling pathways, including the MAPK/ERK and PI3K/AKT pathways, which play extremely important roles in gastric tumorigenesis^5,48,49^. In support of these observations, our data showed that SLC39A10 overexpression significantly increased the phosphorylation of ERK and the phosphorylation of AKT at Ser473 and Ser129 but only slightly increased the phosphorylation of AKT at Thr308 in gastric cancer cells. Next, to assess the effect of intracellular Zn^2+^ homeostasis on the oncogenic roles of SLC39A10, we treated gastric cancer cells with the highly selective Zn^2+^ chelator TPEN and found that Zn^2+^ chelation effectively attenuated the promoting effects of SLC39A10 overexpression on cell proliferation and colony formation. As expected, Zn^2+^ chelation also reversed the activating effect of SLC39A10 overexpression on the MAPK/ERK and PI3K/AKT signaling pathways, further supporting the above conclusions.

It is well known that CK2 is a highly conserved and ubiquitous serine/threonine protein kinase. This kinase can participate in regulating the activity of the MAPK/ERK and PI3K/AKT signaling pathways, and its structure is highly dependent on Zn^2+^ (see refs. ^57,58^). To further explain the important effect of Zn^2+^ on the conformation of CK2, we expressed and purified CK2β proteins and found that Zn^2+^ significantly increased the content of β-sheets in the protein, indicating the formation of a zinc finger structure. Moreover, the results of CD and thermal denaturation assays also demonstrated that the melting temperature (Tm) was elevated in the presence of Zn^2+^, proving that Zn^2+^ can stabilize the CK2β protein structure. In addition, native PAGE and Coomassie blue staining provided direct evidence indicating that the CK2β protein formed a dimeric structure containing Zn^2+^.

The CK2 protein is a structural kinase that can phosphorylate acidic proteins, and a number of substrates have been identified^59^ that regulate a variety of physiological and pathological processes, including proliferation, transformation, apoptosis, senescence and malignant transformation^60^. In addition, the dysregulation of CK2 has been implicated in tumorigenesis in various cancers, including gastric cancer^61,62^, suggesting that it may be a potential therapeutic target in human cancers. Silmitasertib (CX-4945) is a highly selective orally bioavailable small molecule inhibitor of CK2 that shows an excellent kinase selectivity profile and potent antitumor activity against different types of cancer cells, including gastric cancer cell lines^63–65^. At present, CX-4945 has been approved for clinical trials in some human cancers, including basal cell carcinoma, multiple myeloma, medulloblastoma, cholangiocarcinoma, breast cancer and kidney cancer (https://clinicaltrials.gov/). Recently, one of the human phase 2 clinical trials in coronavirus disease 2019 (COVID-19) was completed (NCT04663737). These observations suggest that targeting CK2 constitutes a new strategy for gastric cancer therapy.

Considering the above findings, we speculate that SLC39A10 promotes malignant phenotypes of gastric cancer cells by enhancing the enzyme activity of Zn^2+^-dependent CK2. Indeed, our data demonstrated that SLC39A10 overexpression and knockdown led to a significant increase and decrease in CK2 activity in gastric cancer cells, respectively, and that this effect was effectively reversed by the Zn^2+^ chelator TPEN or CX-4945. Correspondingly, CX-4945 reversed the promoting effect of SLC39A10 overexpression on ERK1/2 and AKT phosphorylation and c-Myc expression. As expected, CX-4945 attenuated the proliferation-promoting effect of SLC39A10 overexpression, further supporting the above conclusions. In addition, we found that SLC39A10 knockdown or overexpression did not change the protein and mRNA expression of CK2α. However, SLC39A10 knockdown and overexpression significantly downregulated and upregulated, respectively, the protein expression of CK2β but did not affect its mRNA expression. Further studies demonstrated that SLC39A10 knockdown decreased CK2β protein stability via the proteasomal degradation pathway, thereby suppressing the holoenzyme activity of CK2. The above findings indicate that SLC39A10 promotes malignant phenotypes of gastric cancer cells by activating Zn^2+^-dependent CK2-mediated MAPK/ERK and PI3K/AKT pathways, while c-Myc, as a major downstream effector of these two pathways, directly transcriptionally regulates SLC39A10, forming a vicious feedback loop and then accelerating the malignant progression of gastric cancer.

In summary, we found that elevated expression of SLC39A10 is common in gastric cancers and is strongly related to poor patient outcomes. Through a series of in vitro and in vivo functional experiments, we demonstrated that SLC39A10 forms a vicious loop with c-Myc to exert its oncogenic roles by promoting Zn^2+^ efflux and subsequently activating the CK2-mediated MAPK/ERK and PI3K/AKT signaling pathways. Taken together, these data indicate that SLC39A10 is a potential target for gastric cancer therapy, and targeting CK2 may constitute an alternative therapeutic strategy for gastric cancer patients with high SLC39A10 expression.

## Supplementary information

Supplementary Materials
