# Supplementary material for: SLC39A10 promotes malignant phenotypes of gastric cancer cells by activating the CK2-mediated MAPK/ERK and PI3K/AKT pathways
Source: Exp Mol Med. 2023 Aug 1;55(8):1757–69. doi: 10.1038/s12276-023-01062-5 (PMC10474099; doi:10.1038/s12276-023-01062-5)
Supplement: Supplementary file 1 — Supplementary Materials [file 12276_2023_1062_MOESM1_ESM.pdf]

Supplementary Materials

Supplementary Table 1. qRT-PCR primers used in this study

Genes	Forward primer (5'-3')	Reverse primer (5'-3')
SLC39A10	GTGCTGGATTGACAGGAGGAA	GACCAACAGCTGTGCCTATGA
CSNK2B	GCCTTTCAGACATCCCAGGT	ACTGGTTGGCAGGTCTCTTG
c-Myc	CACCGAGTCGTAGTCGAGGT	TTTCGGGTAGTGGAAAACCA
18S	CGCCGCTAGAGGTGAAATTC	CTTTCGCTCTGGTCCGTCTT
β-actin	CCTTGCACATGCCGGAG	GCACAGAGCCTCGCCTT

Supplementary Table 2. siRNAs and shRNAs used in this study

siRNAs	Sequence (5'-3')
si-SLC39A10-#1	CCACAAACCTGATCGTGTA
si-SLC39A10-#3	ACAGCATCGTGGAATGACA
si-NC	UUCUCCGAACGUGUCACGUTT
si-c-Myc	AACGATTCCTTCTAACAGA
sh-NC (for sh-SLC39A10)	GATCCGTTCTCCGAACGTGTCACGTAATTCAA GAGATTACGTGACACGTTCGGAGAATTTTTTC
sh-SLC39A10	GATCCGACAGCATCGTGGAATGACATTCAAGA GATGTCATTCCACGATGCTGTTTTTTTG

Supplementary Table 3. Antibodies used in this study

Antibodies	Catalog#	Source
anti-SLC39A10	NBP1-76507	Novus
phospho-CK2 Substrate [(pS/pT)DXE]	#8738	Cell Signaling Technology
anti-Ki-67	550609	BD Pharmingen
anti-ERK1/2	#4695	Cell Signaling Technology
anti-phospho-ERK1/2	#4370	Cell Signaling Technology
anti-phospho-AKT (Ser129)	#13461	Cell Signaling Technology
anti-total AKT	BS1810	Bioworld Technology
anti-phospho-AKT (Ser473)	BS4007	Bioworld Technology
anti-phospho-AKT (Thr308)	BS4009	Bioworld Technology
anti-CK2α	10992-1-AP	proteintech
anti-CK2β	ab76025	abcam
anti-c-Myc for WB	sc-764	Santa Cruz
anti-c-Myc for IHC	sc-40	Santa Cruz
anti-β-Actin	sc-1616	Santa Cruz

Supplementary Table 4. The primers used in this study for luciferase reporter plasmid construction

Construct	Position	Forward primer (5'-3')	Reverse primer (5'-3')	Restriction sites
pGL3-SLC39A10-Luc	Promoter (-1997/+10)	TCTATCGATAGGTACCCAGCCTTTTTTCAGTTTTGACTGTG	GATCGCAGATCTCGAGGTACCTTCATTTCTATTTTTCCTAAAGAG	*Kpn I* & *Xho I*

Supplementary Table 5. The primers used in this study for ChIP assay

Gene	Position	Forward primer (5'-3')	Reverse primer (5'-3')
SLC39A10	P1: -1044/-941	TGATGGACATGTTCTGCGTC	AAATGCAGCCTCTCAGTCAC
	P2:-1518/-1273	AGTCTTCTCCCAGTCTGCAG	CACTTACAGCACATCCTAATTCAG
	P3: -1939/-1671	CATGTACATGGATTTCTGTTGG	GCTCAGAAGTCTTCAGGAAACC

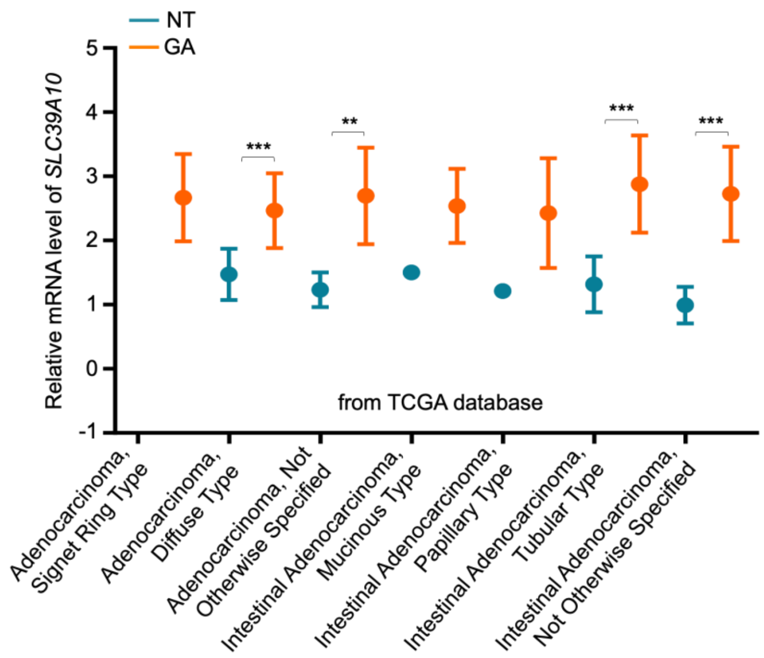

Supplementary Fig. 1 The mRNA expression of *SLC39A10* in different histologic types of gastric cancer using TCGA database. GA, gastric adenocarcinoma; NT, normal gastric tissue; **, *P* <0.01; ***, *P* <0.001.

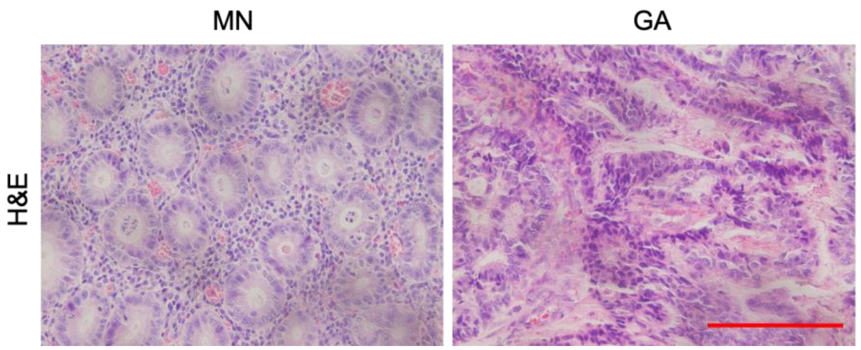

Supplementary Fig. 2 Representative images showing H&E staining on histologic slides of gastric adenocarcinoma (GA) and matched normal gastric tissue (MN). Scale bar, 200 μm.

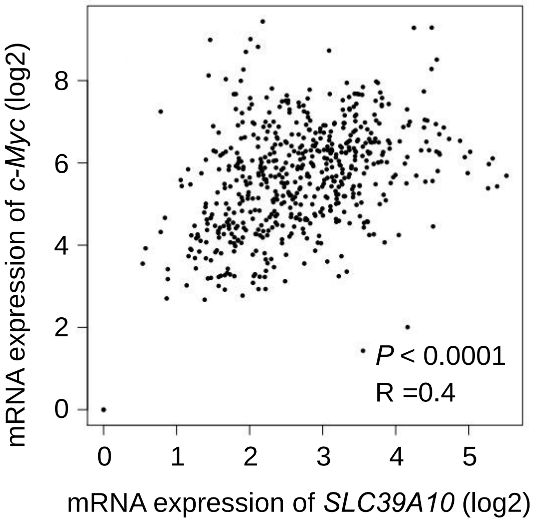

Supplementary Fig. 3 Linear regression analysis showing the association of mRNA expression of *SLC39A10* with mRNA expression of *c-Myc* (*R* =0.4; *P* <0.0001) in gastric cancers (the data from the TCGA database and GTEx database).

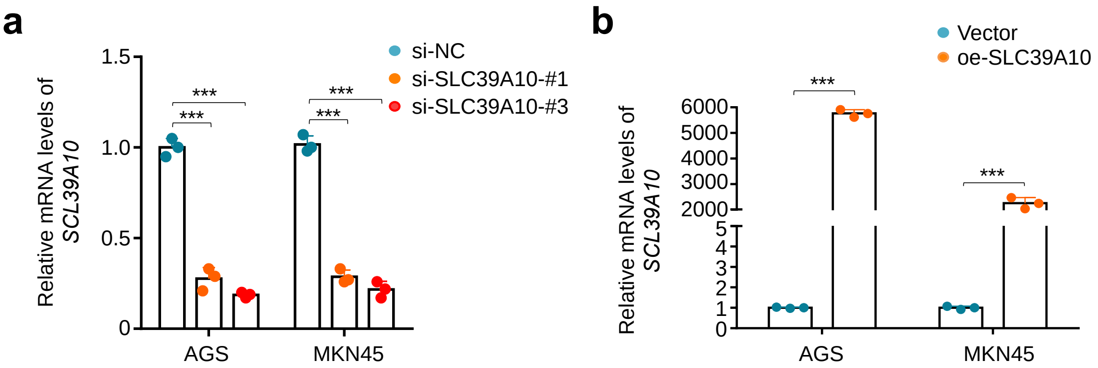

Supplementary Fig. 4 a SLC39A10 knockdown by two different siRNAs (si-SLC39A10-#1 and -#3) in AGS and MKN45 cells was confirmed by qRT-PCR assay. **b** Ectopic expression of SLC39A10 was also confirmed by qRT-PCR assay. *β-actin* was used as a reference gene. ***, *P* <0.001.

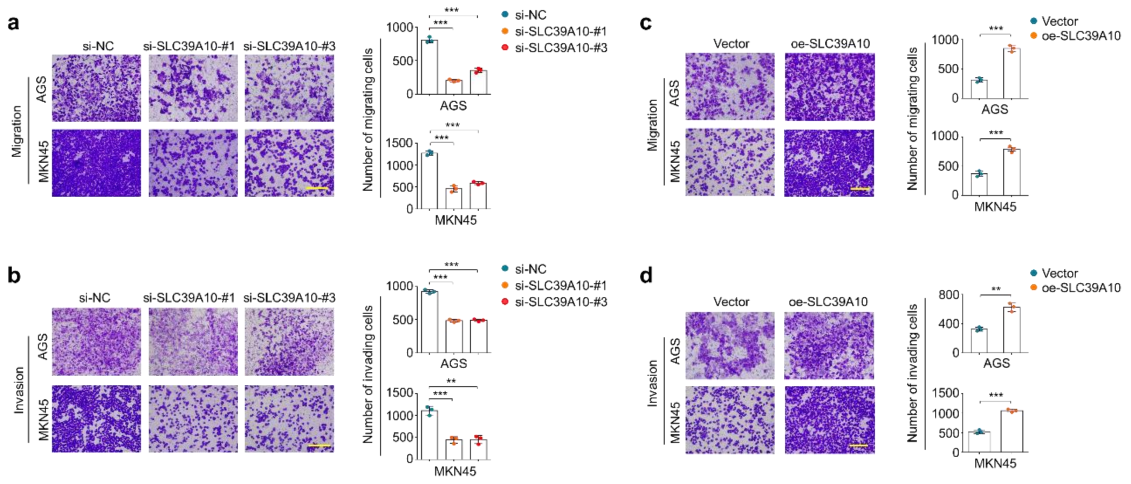

Supplementary Fig. 5 Inhibitory effect of SLC39A10 knockdown on gastric cancer cell migration (**a**) and invasion (**b**). Promoting effect of ectopic expression of SLC39A10 in AGS and MKN45 cells on gastric cancer cell migration (**c**) and invasion (**d**). Left panels represent representative images of migrating/invaded cells. Quantitative illustration of cell numbers is shown as the means ± SDs in the right panels. **, *P* <0.01, ***, *P* <0.001.

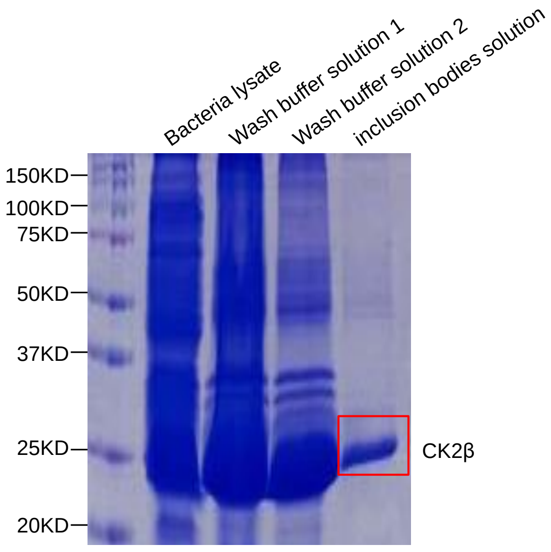

Supplementary Fig. 6 SDS-PAGE and coomassie blue staining proving successful expression and purification of CK2β (red box).

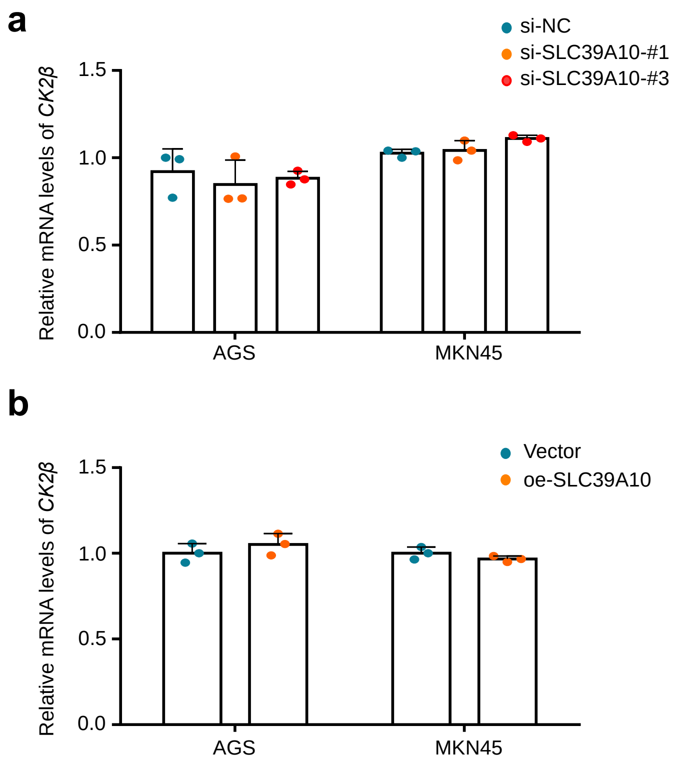

Supplementary Fig. 7 RT-PCR analysis was performed to evaluate the effect of SLC39A10 knockdown (**a**) and overexpression (**b**) in AGS and MKN45 cells on mRNA expression of *CK2β*. *β-actin* was used as a reference gene.

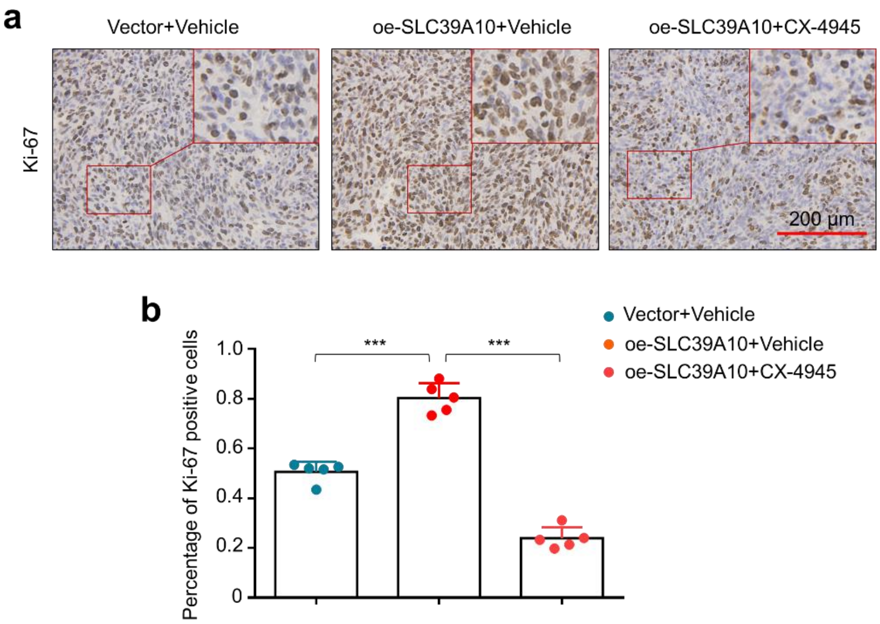

Supplementary Fig. 8 a Shown is representative Ki-67 staining of xenograft tumors from the indicated groups. **b** Histogram represents means ± SDs of the percentage of Ki67 positive cells from five microscopic fields in each group. Scale bar, 200 µm. ***, P <0.001.

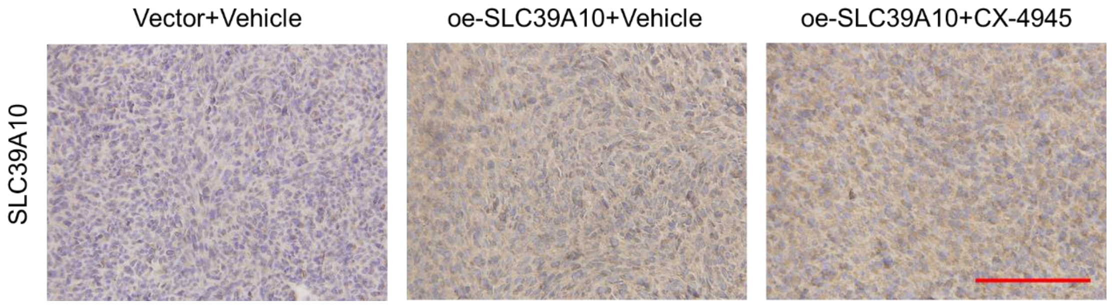

Supplementary Fig. 9 Shown is representative SLC39A10 staining of SLC39A10-overexpressing xenograft tumors and control tumors with the indicated treatments. Scale bar, 200 µm.

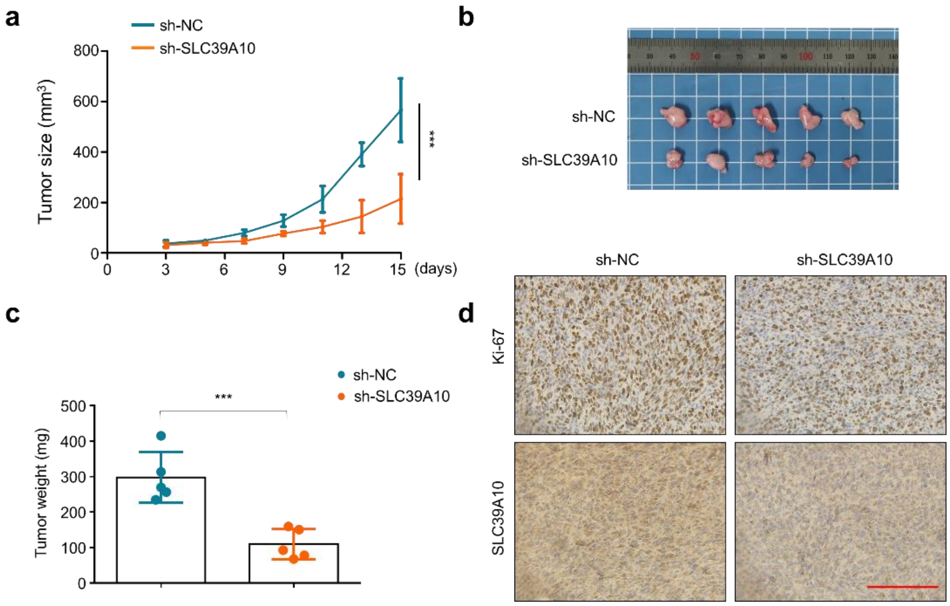

Supplementary Fig. 10 a Growth curves of SLC39A10-knockdown xenograft tumors and control tumors (n =5/group). **b** The photographs of dissected tumors from the indicated groups. **c** Mean tumor weight from the indicated groups. **d** Representative Ki-67 and SLC39A10 staining of xenograft tumors from the indicated groups. Scale bar, 200 μm. sh-NC, control shRNA; sh-SLC39A10, shRNA targeting SLC39A10; ***, *P* <0.001.

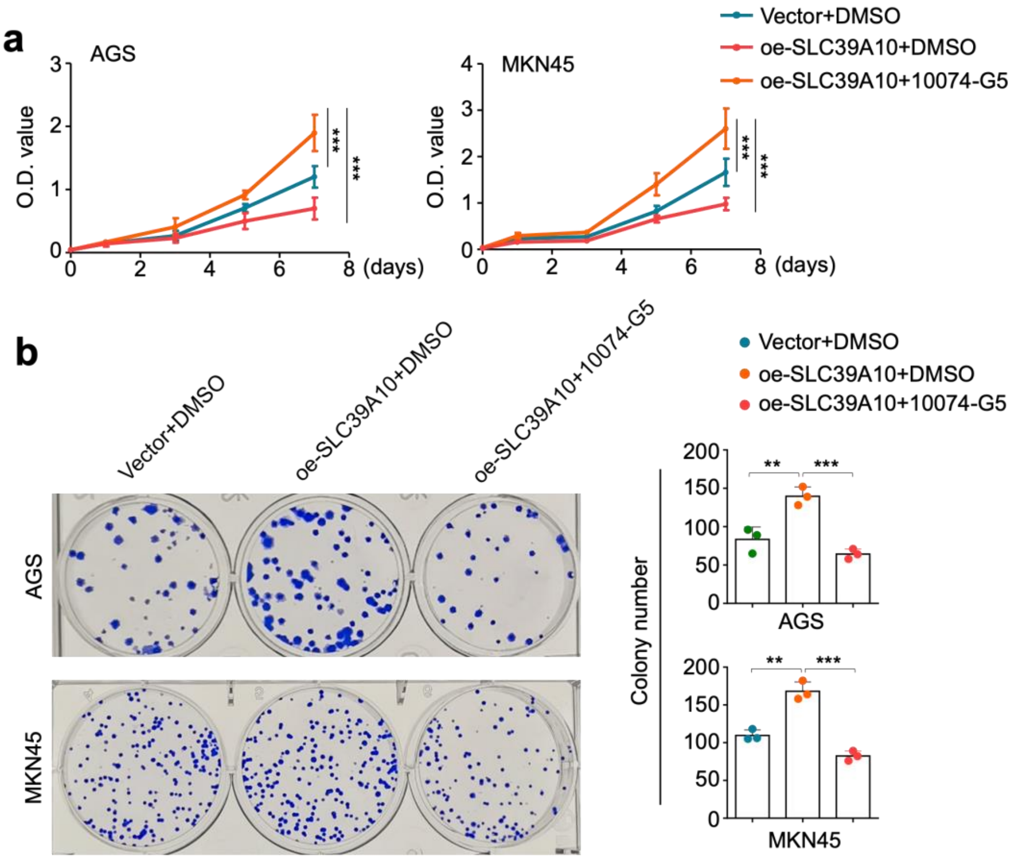

Supplementary Fig. 11 a SLC39A10-overexpressing AGS and MKN45 cells and control cells were treated with10 μM 10074-G5 or vehicle for 24 h, and the effects of these treatments on cell proliferation was evaluated by MTT assay. **b** The colony formation assay in AGS and MKN45 cells with the indicated treatments. Representative images of colony formation assays in the left panels, and quantitative analysis was shown in the right panel. The data are presented as the means ± SDs. **, P <0.01; ***, P <0.001
